# Optimizing post-operative imaging: a retrospective cohort study comparing two methods of lateral hip radiography after cephalomedullary nail surgery

**DOI:** 10.1186/s12891-023-06495-7

**Published:** 2023-05-09

**Authors:** Da Huang, Gui-Yue Chen, Hui Liu, Hai-Wen Cui, Li-Xin Wang, Yu-Jing Chen, Xi-Shuai Yang

**Affiliations:** 1 Department of Orthopaedic Surgery, Changzhi People’s Hospital, The Third Affiliated Hospital of Changzhi Medical College, No. 502 of Changxing Middle Road, Luzhou District, Changzhi, 046000 Shanxi China; 2 Department of Medical Radiology, Changzhi People’s Hospital, The Third Affiliated Hospital of Changzhi Medical College, No. 502 of Changxing Middle Road, Luzhou District, Changzhi, 046000 Shanxi China; 3 Department of Neurology, Changzhi People’s Hospital, The Third Affiliated Hospital of Changzhi Medical College, No. 502 of Changxing Middle Road, Luzhou District, Changzhi, 046000 Shanxi China

**Keywords:** Hip fracture, Proximal femur fracture, Cephalomedullary nail (CMN), Lateral radiograph, Lateral X-ray, Lateral X-ray imaging

## Abstract

**Background:**

Currently, there is no consensus on the most appropriate technique for obtaining lateral hip radiographs after cephalomedullary nail (CMN) surgery. The aim of this study was to investigate the distribution of two commonly used postoperative lateral hip radiographic methods (classic lateral view and modified lateral view) and try to find out which one is better suited for this situation.

**Methods:**

A retrospective analysis was conducted on 146 patients who underwent surgical fixation for extracapsular hip fractures between January 2018 and June 2022. The main outcome measured was the angle between the straight part of the CMN and the lag screw/blade on hip lateral X-rays (CMNA). The lateral hip radiographs were categorized into two groups based on different lateral hip radiographic methods. CMNA, patient age, gender, fracture classification based on the 2018 AO classification, nail length (short/long), surgical side (left/right), height, weight, BMI, preoperative waiting time, postoperative imaging interval were collected and compared between the two groups.

**Results:**

The distribution trend of CMNA significantly differs between two types of hip joint lateral radiographic methods. Specifically, the classic lateral method exhibits a significantly bimodal and skewed distribution with a median (p25, p75) of -21.6° (-31.2°, -8°), whereas the modified lateral method presents a normal distribution with a mean ± SD of +7.57° ± 14.4°. The difference in the Mean Rank between the classic (47.10) and the modified (102.96) lateral methods is statistically significant (P < 0.001).

**Conclusions:**

The CMNA method is an excellent tool for studying the lateral distribution.We recommend using the modified lateral view as the preferred option for obtaining lateral hip radiographs after CMN surgery due to its superior distribution of CMNA and greater patient-friendliness.

**Supplementary Information:**

The online version contains supplementary material available at 10.1186/s12891-023-06495-7.

## Background

Hip fractures in older adults pose a significant global public health challenge [1–7]. Cephalomedullary nails (CMN) have become the preferred treatment for intertrochanteric and subtrochanteric fractures [8, 9], with ideal fracture reduction and lag screw/blade positioning being critical for successful surgery [10]. Although C-arm/fluoroscopic guidance is used for reduction and screw/blade insertion, the imaging resolution, scope, and network printing are limited, necessitating postoperative radiographs. In addition, an ideal postoperative lateral hip radiographs are essential for demonstrating surgical outcomes and providing accurate data for future research based on TAD etc.

**Fig. 1:**
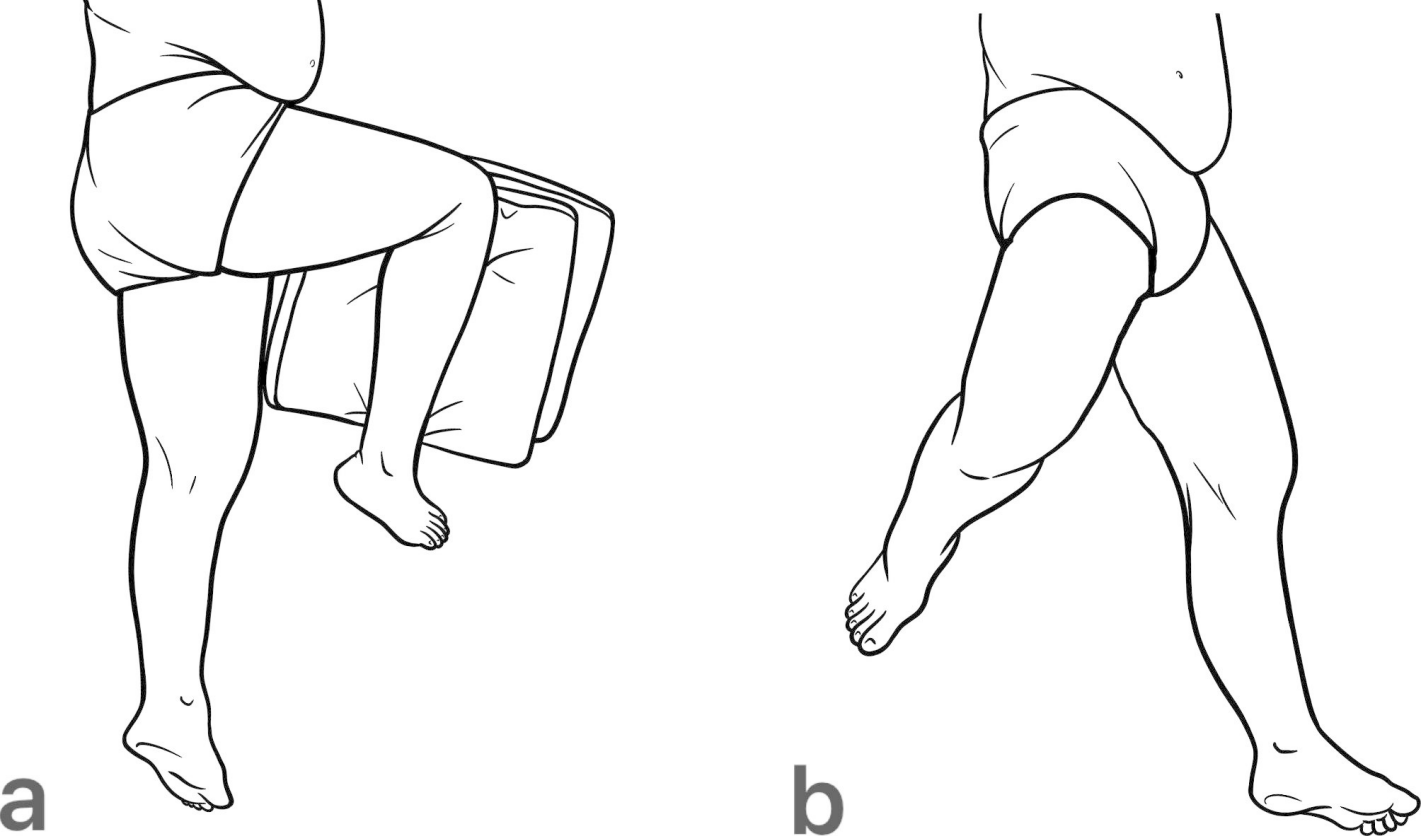
The two most commonly used patient postures for lateral hip radiographs (left side). **a**: the classic lateral view **b**: the modified lateral view. Our radiographers use a 35° cephalic tube angle to obtain the images

Various methods have been developed for taking lateral hip radiographs [11–14], but there is no consensus on the most appropriate technique after cephalomedullary nail (CMN) surgery. Lateral hip radiographs can vary significantly because it require patients to maintain a special posture, which can be difficult for elderly patients after surgery. Additionally, the multiple methods for postoperative lateral X-ray imaging techniques may further increase the variability of postoperative lateral X-rays. The aim of this study is to investigate the distribution of two commonly used postoperative lateral hip radiographic methods: classic lateral view and modified lateral view (Fig. 1) and try to find out which one is better suited for this situation. Accurately measuring and analyzing lateral variations is crucial in this area, and our research provides a novel solution.

## Methods

With institutional review board approval, we finally retrospectively reviewed 146 consecutive patients who underwent surgery for extracapsular hip fractures at our institution between January 2018 and June 2022. The most commonly used cephalomedullary nails (CMNs) in our institution are the proximal femur nail anti-rotation (PFNA-II/Asian version) [15, 16].

**Fig. 2:**
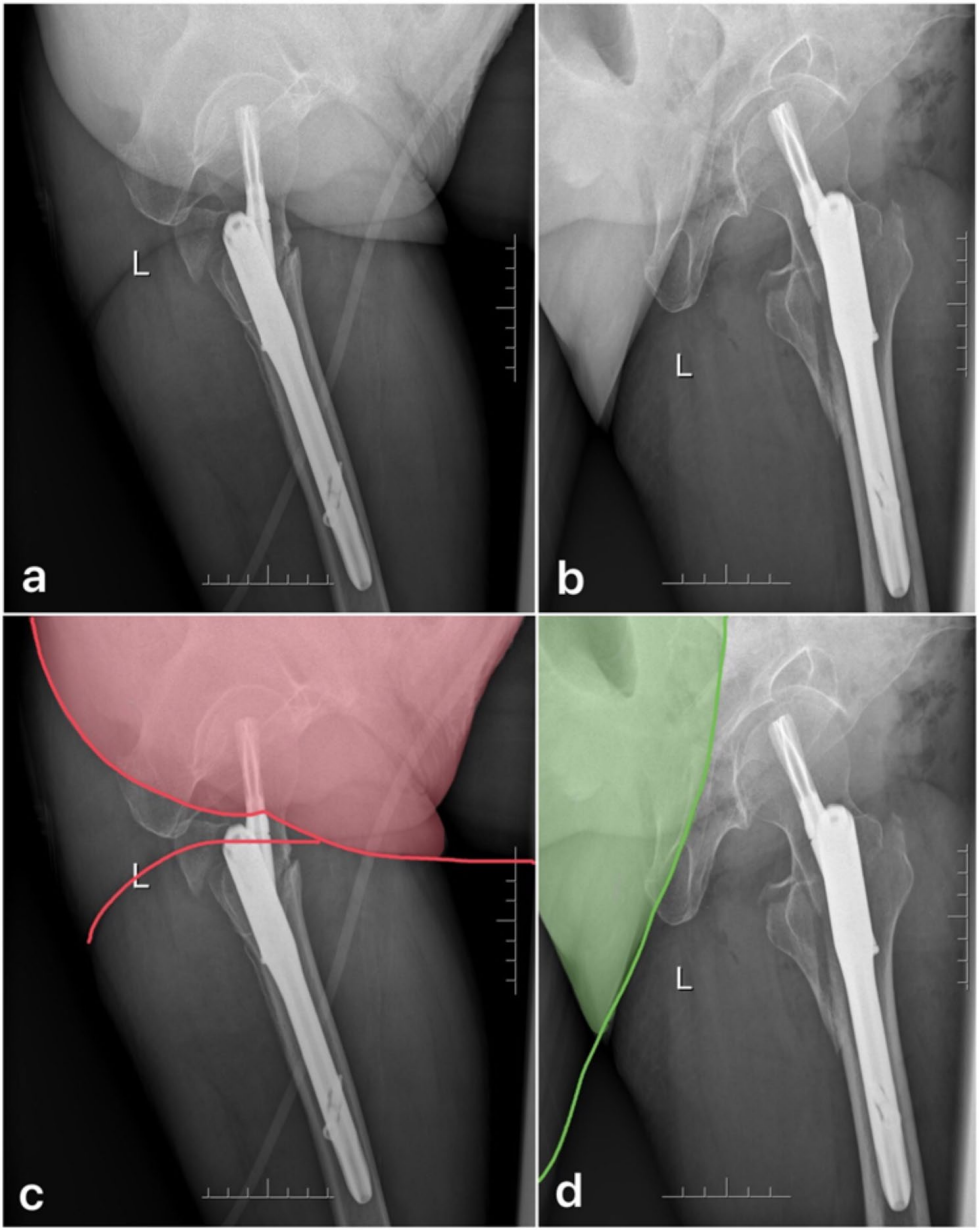
Categorization of lateral hip X-rays. **a**: Classic lateral view **b**: Modified lateral view **c/d**: In these figures, red and green lines represent unique skin patterns, while light red and light green areas indicate areas of overlap and increased density. It should be noted that comparing postoperative AP X-rays taken at the same time can also aid in making a judgment

Inclusion criteria:1) Patients with extracapsular hip fractures, including intertrochanteric or subtrochanteric fractures; 2) Lateral hip radiographs taken after surgery and before discharge; 3) Age 55 years or older; 4) Patients treated with PFNA. Exclusion criteria: 1) Patients with polytrauma injury; 2) Uncertainty or disagreement in classifying the lateral X-rays imaging technique; 3) Poor quality X-rays that cannot be measured. Radiographers are experienced team members who took lateral hip radiographs based on their personal experience and preference. One radiographer and one orthopedic trauma surgeon classified the lateral hip radiographs into two categories based on the soft tissue shadow of the buttocks and legs (Fig. 2).

**Fig. 3:**
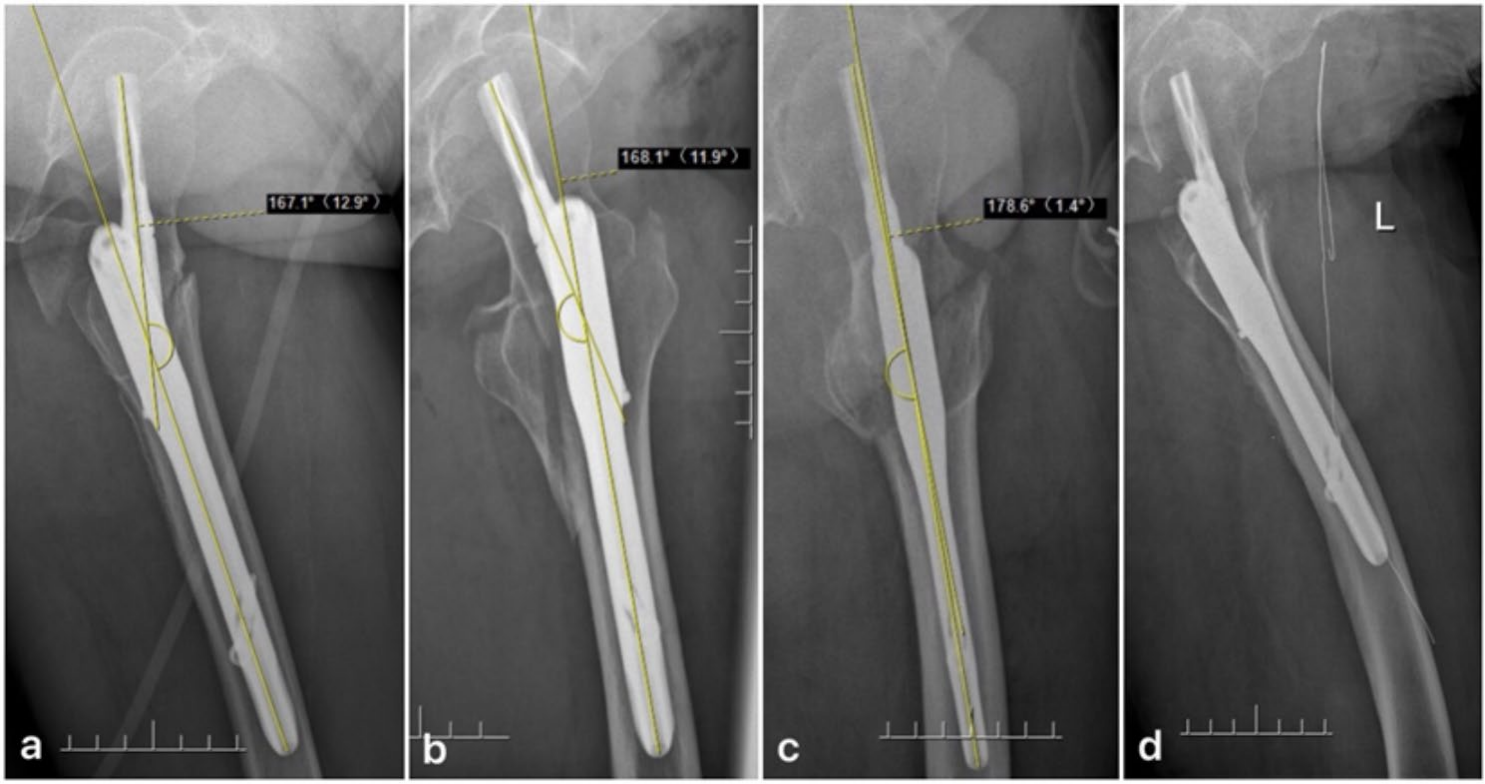
**a**: CMNA = -12.9°. **b**:CMNA = + 11.9°. **c**: “Ideal radiograph” showing CMNA = +1.4°. As the hip region is asymmetrical in the anteroposterior direction, a positive value was assigned when the helical blade was oriented in the same direction as the AP view, and a negative value was assigned when it was oriented in the opposite direction. **d**: The presence of the femoral bow and the obscuration of the femoral neck by the CMN make measurements such as NSA unreliable

The main focus of this study was to measure the angle between the straight part of the cephalomedullary nail (CMN) and the blade/head screw on lateral hip X-rays, a unique feature of the CMN structure. This angle was named “CMNA” and is shown in Fig. 3.

Although the neck-shaft angle (NSA), collodiaphyseal angle (CDA), and caput-collum-diaphyseal (CCD) angle can also be used to describe the distribution of different lateral hip views, CMNA was chosen for its objectivity, conciseness, and accuracy. All CMNA measurements were taken using the protractor APP provided with the PACS (picture archiving and communication system). For longer intramedullary nails (> 240 mm), which have an anterior arch at the distal end, only the proximal straight part were measured. Some tips for measuring CMNA include zooming in, adjusting contrast if necessary, and referencing the tail and hollow axis of the blade when measuring the blade axis. For the main nail axis, we suggest considering the abduction of the PFNA near the proximal end and using the hollow axis of the straight part of the main nail as the reference. In order to test the reliability of CMNA, in addition to CMNA1 (initial data by Da Huang), we also added CMNA2 (by Da Huang), CMNA3 (by Li-Xin Wang), and CMNA4 (by Li-Xin Wang), and the average of the four sets of numerical values was used in this study.

The study collected data on the CMNA, patient age, gender, fracture classification based on the 2018 AO classification, nail length (short/long), surgical side (left/right), height, weight, BMI, preoperative waiting time, postoperative imaging interval, and type of lateral shooting method.

The radiographs of all patients with extracapsular hip fractures were reviewed by two senior orthopaedic trauma surgeons and categorized into 31A1, 31A2, 31A3, and subtrochanteric types according to the 2018 OTA/AO classification.

### Statistical analysis

The comparison of CMNA between the classic and modified lateral positions will be performed using the Mann-Whitney U test to determine if there is a significant difference between the two positions. Age will be compared using the t-test. Gender, fracture classification, nail type (short/long), and side (left/right) will be compared using the chi-square test. Height, weight, BMI, preoperative waiting time, and postoperative to imaging interval will be compared using the Mann-Whitney U test. We also conducted CMNA inter- and intra- correlation tests. A P-value of ≤ 0.05 was considered significant. Data were analyzed using SPSS version 28.0.

## Results

A total of 224 consecutive extracapsular hip fracture patients from 2018.01 to 2022.06 were included in the study, with 21 cases excluded due to the lack of postoperative lateral hip radiographs, 43 cases excluded due to the prescription of lateral femur radiographs, 9 cases excluded due to age younger than 55 years, 1 case excluded due to multiple injuries, 2 cases excluded due to internal fixation other than PFNA, and 2 cases excluded due to different opinions on the classification of lateral hip radiographic methods. Finally, 146 cases met our inclusion and exclusion criteria. The detailed information on patient demographics, baseline characteristics and CMNA can be found in Table 1.

**Table 1:** Patient demographics, baseline characteristics and CMNA

Variable	classic lateral view(n = 77)	modified lateral view(n = 69)	p value
**Age, mean ± SD, (y)**	76.09 ± 8.412	75.71 ± 8.573	0.787
**Gender, n (%)**			0.622
**Gender, n (%)** **Male** **Female**	26(33.8%) 51(66.2%)	26(37.7%) 43(62.3%)	
**OTA/AO class, n (%)**			0.551
**A1** **A2** **A3** **Subtrochanteric**	2(2.6%) 59(76.6%) 13(16.9%) 3(3.9%)	5(7.2%) 53(76.9%) 9(13%) 2(2.9%)	
**Nail type, n (%)**			0.798
**Short** **Long**	67(87.0%) 10(13.0%)	61(88.4%) 8(11.6%)	
**Side, n (%)**			0.053
**Left** **Right**	48(62.3%) 29(37.7%)	32(46.4%) 37(53.6%)	
**Height,(cm)**			0.242
	N = 65 Median(p25,p75) 160(155,166) Mean Rank 61.19	N = 64 Mean ± SD 162.11 ± 7.91 Mean Rank 68.87	
**Weight,(kg)**			0.127
	N = 65 Median(p25,p75) 55(0,60)5 Mean Rank 60.04	N = 64 Mean ± SD 59.88 ± 11.18 Mean Rank 70.04	
**BMI,(kg/m**^**2**^)			0.042
	N = 65 Median(p25,p75) 20.96(19.71,23.59) Mean Rank 58.35	N = 64 Mean ± SD 22.92 ± 3.73 Mean Rank 71.76	
**Preoperative waiting time median,(d)**			0.930
	Median(p25,p75) 3.00(2,6) Mean Rank 73.79	Median(p25,p75) 3.00(2,5) Mean Rank 73.18	
**Postoperative to imaging interval median, (d)**			0.086
	Median(p25,p75) 2.00(2,3) Mean Rank 78.81	Median(p25,p75) 2.00(1,2) Mean Rank 65.57	
**CMNA,(°)**			<0.001
	Median(p25,p75) -21.6(-31.2,-8) Mean Rank 47.10	Mean ± SD +7.57 ± 14.4 Mean Rank 102.96	

We noticed that there were no differences in age, gender, OTA/AO class, nail type (short/long), height, weight, and preoperative waiting time between the two groups.

However, there was a significant difference in BMI between the two groups of patients. In the classic lateral view group (N = 65), the median (p25, p75) BMI was 20.96 (19.71, 23.59) and the mean rank was 58.35. In the modified lateral view group (N = 64), the mean ± SD BMI was 22.92 ± 3.73, and the mean rank was 71.76. The difference between the two groups was significant (P = 0.042).

The surgery side distribution of left and right in classic lateral position were 48 (62.3%) on the left and 29 (37.7%) on the right, and in modified lateral position were 32 (46.4%) on the left and 37 (53.6%) on the right. The p-value is 0.053, which were almost marginally significant.

The time interval between surgery and the X-ray examination seemed to have had a potential influence on the choice of X-ray views. In the classic lateral view group, the median (p25, p75) time interval was 2.00 (2, 3), and the mean rank was 78.81. In the modified lateral view group, the median (p25, p75) time interval was 2.00 (1, 2), and the mean rank was 65.57. The difference was possibly significant (P = 0.086).

**Fig. 4:**
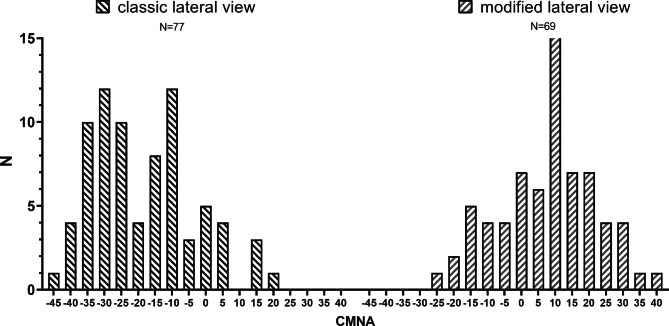
This image reveals the distribution of CMNA in two different shooting methods. It is noteworthy that the CMNA distribution in the classic lateral view was found to deviate from a normal distribution. Furthermore, the distribution of CMNA was observed to vary dramatically between the two X-ray imaging methods, the modified lateral position is closer to the ideal position (CMNA=0°) compared to the classic lateral position.

The distribution trend of CMNA significantly differs between two types of hip lateral radiographic methods. Specifically, the classic lateral method exbited a significantly bimodal and skewed distribution with a median (p25, p75) of -21.6° (-31.2°, -8°), whereas the modified lateral method presented a normal distribution with a mean ± SD of +7.57° ± 14.4°. The difference in the Mean Rank between the classic (47.10) and the modified (102.96) lateral methods was statistically significant (P < 0.001). Please refer to Fig. 4 for specific distribution details.

The results of the reliability analysis (intra- and inter-rater) of CMNA was almost 100%.

## Discussion

Postoperative standard hip lateral radiographs are necessary to confirm the fracture reduction with the three-grade classification system [17] and the position of the head screw/blade in the femoral head using various methods, such as Cleveland’s method [18], Parker’s ratio index [19], Tip-apex distance (TAD) [17], calcar referenced tip-apex distance (Cal TAD) [20], and Angle DAE [21]. These radiographs and evaluations can provide reassurance and validation for the department head, colleagues, and patients. In addition, variation in postoperative lateral hip X-ray imaging can lead to inaccurate measurements of indicators such as TAD, compromising the reliability of studies on head screw/blade cut-out risk factors. 

The variability in postoperative lateral hip X-ray imaging after CMN surgery is a concern in clinical practice, as evidenced by the poor quality of radiographs commonly observed in academic exchanges. The reasons for this variability may include difficulties that elderly patients face in maintaining a specific position during imaging and the use of different lateral radiographic techniques. Therefore, it is crucial to ascertain whether both imaging techniques are equally effective or identify the most suitable imaging technique for this particular scenario.However, the lack of reliable evaluation methods and sufficient data has made this challenging. Our research has addressed this gap by providing a novel and reliable measurement method along with rich data.

**Fig. 5:**
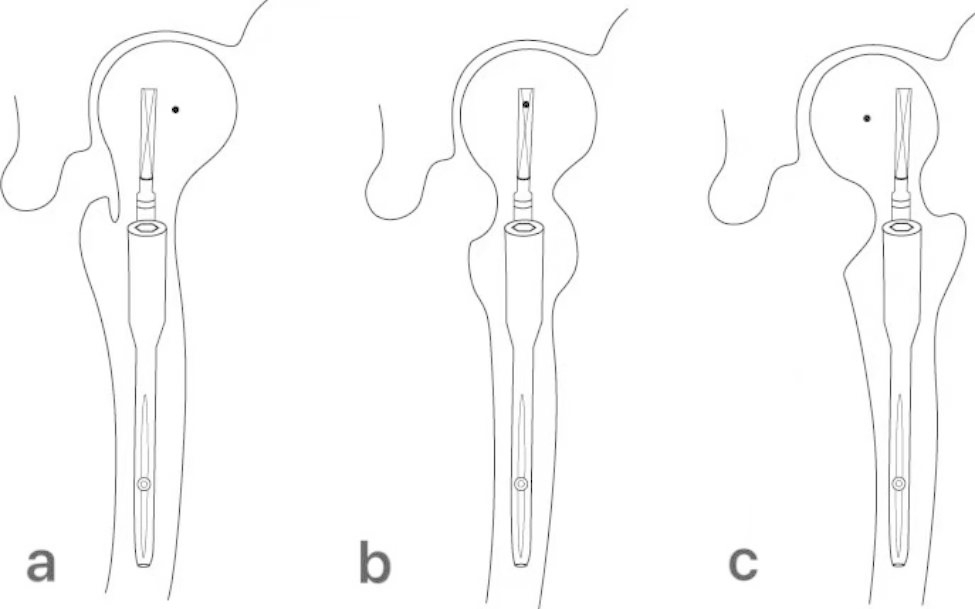
A new head screw/blade position system. **a**: located at the posterior aspect. **b**: located at the middle aspect. **c**: located at the anterior aspect

We suggest that CMNA is an excellent method for studying lateral distribution. The simplicity of the CMNA measurement method is a significant advantage, and our testing shows that the reliability analysis of CMNA is nearly 100%. A CMNA value of 0° corresponds to the standard intraoperative procedure of confirming the fracture reduction in the true lateral position of the femoral head and neck and placing the head screw/blade in the center of the femoral head. Essentially, CMNA = 0° indicates the true lateral view of the femoral head and neck that has been confirmed intraoperatively through the C-arm. While it is more suitable for intraoperative use, for postoperative radiographic review, we propose a new head screw/blade position system (Fig. 5) based on CMNA = 0° instead of the true lateral position of the femoral neck.

Although this study is retrospective, we aimed to ensure comparability of the baseline or demonstrate possible selection bias/patient preference by comparing age, gender, OTA/AO class, nail type (short/long), side (left/right), height, weight, preoperative waiting time, and postoperative to imaging interval between the two groups.Here were our findings: 1) There were no differences between the groups in terms of age, gender, OTA/AO class, nail type (short/long), height, weight, and preoperative waiting time. 2) However, the BMI of the two groups showed a significant difference(P = 0.042), with patients in the modified lateral view group having a higher mean BMI and rank. We discussed this finding with our radiologic technologists and concluded that patients with higher BMI tend to receive the modified lateral view because it is easier for them to adopt this position. 3) Our study also found that prior surgery on the left or right hip may influence the selection of imaging position. The distribution of classic and modified lateral positions varied almost marginally significant (P = 0.053) between left and right hips, with the modified lateral position being more adaptable and suitable for both sides. We hypothesized that this was because the classic lateral position required sufficient strength in the contralateral leg (most people are right-handed/legged) to enable complete trunk rotation, whereas the modified lateral position only necessitates partial trunk rotation, which can be achieved with basic strength. 4) In addition, our study suggests that the interval between surgery and radiography may potentially influence the choice of imaging method. Although the results are not statistically significant(P = 0.086), we found that patients might have preferred the modified lateral position when the time interval between surgery and radiography was short, as it might have exerted less pressure/pain on the surgical site than the classic lateral position. 5) Overall, our findings highlight the importance of considering patient characteristics when selecting an imaging position, and suggest that the modified lateral position may be a more versatile option in certain situations. However, further experiments are needed to validate these findings.

Our study confirmed significant differences (P<0.001) in the distribution of CMNA between two lateral hip radiography methods. This indicates that the choice of projection method is an important factor contributing to the significant variation in lateral hip X-ray imaging after CMN surgery. The observed differences in clinical practice are not simply due to random variations in a normal distribution.

The classic lateral view had a significantly bimodal and skewed distribution, with a Median(p25,p75) of -21.6°(-31.2°,-8°). The median value was significantly deviated from CMNA = 0°, and the distribution had a high degree of variability. In recent years, there has been increasing attention to anteromedial cortex-to-cortex support reduction [22]. The classic lateral view seems to provide a good observation of this, but we should focus on evaluation of anteromedial cortex reduction quality and correct it during surgery [23]. Achieving CMNA = 0° in the classic lateral view is challenging due to the patient’s unstable position, which easily causes them to fall backward. Therefore, patients tend to tilt forward to achieve stability, resulting in a skewed distribution.

The modified lateral view showed a normal distribution, with a mean ± SD of +7.57°±14.4°. The mean value was very close to CMNA = 0°, indicating that the modified lateral view is a more suitable projection method. In fact, we have already observed the benefits of the modified lateral position in our clinical practice, and our research findings have further validated our initial hypothesis. However, we cannot ignore the notable variability in the modified lateral view. The position of the proximal femur is influenced by the rotation of the target limb and the rolling of the trunk. Assisting the patient in rotating the trunk or externally rotating the leg may further reduce this variability resulting in a closer approximation to the ideal lateral position.

Our study has some limitations: (1) The best study design may be prospective randomized controlled studies with larger sample sizes to further confirm our findings. (2) Many hip imaging methods inherently lack precise definitions or cannot be accurately implemented, which may introduce errors. (3) Some patients did not have lateral radiographs or only had lateral femoral radiographs after surgery. Although these cases were excluded, this may lead to selection bias. (4) Considering the geometrical mismatch between the antecurvation of the femur and the contemporary intramedullary nails in Asians [24], in the future, even short nails may have an anterior curvature design [25, 26], which may pose challenges for the measurement of the CMNA.

## Conclusions

In conclusion, Our suggestion is that the CMNA method is an excellent tool for studying the lateral distribution, and we propose a new head screw/blade position system based on a CMNA angle of 0°. We recommend using the modified lateral view as the preferred option for obtaining lateral hip radiographs after CMN surgery due to its superior distribution of CMNA and greater patient-friendliness.

## Electronic supplementary material

Below is the link to the electronic supplementary material.

Supplementary Material 1

## Data Availability

The datasets used and/or analysed during the current study are available from the corresponding author on reasonable request. The data are not publicly available duo to privacy or ethical restrictions.

## Abbreviations

CMN: Cephalomedullary nail
PFNA: Proximal femur nail anti-rotation
NSA: Neck-shaft angle
CDA: Collodiaphyseal angle
CCD angle: Caput-collum-diaphyseal
PACS: Picture archiving and communication system
