# Supplementary material for: Optimizing post-operative imaging: a retrospective cohort study comparing two methods of lateral hip radiography after cephalomedullary nail surgery
Source: BMC Musculoskelet Disord. 2023 May 9;24:364. doi: 10.1186/s12891-023-06495-7 (PMC10169506; doi:10.1186/s12891-023-06495-7)
Supplement: Supplementary file 1 — Supplementary Material 1 [file 12891_2023_6495_MOESM1_ESM.docx]

Fig.1 the two most commonly used patient postures for lateral hip radiographs (left side). a: the classic lateral view b: the modified lateral view. Our radiographers use a 35° cephalic tube angle to obtain the images.
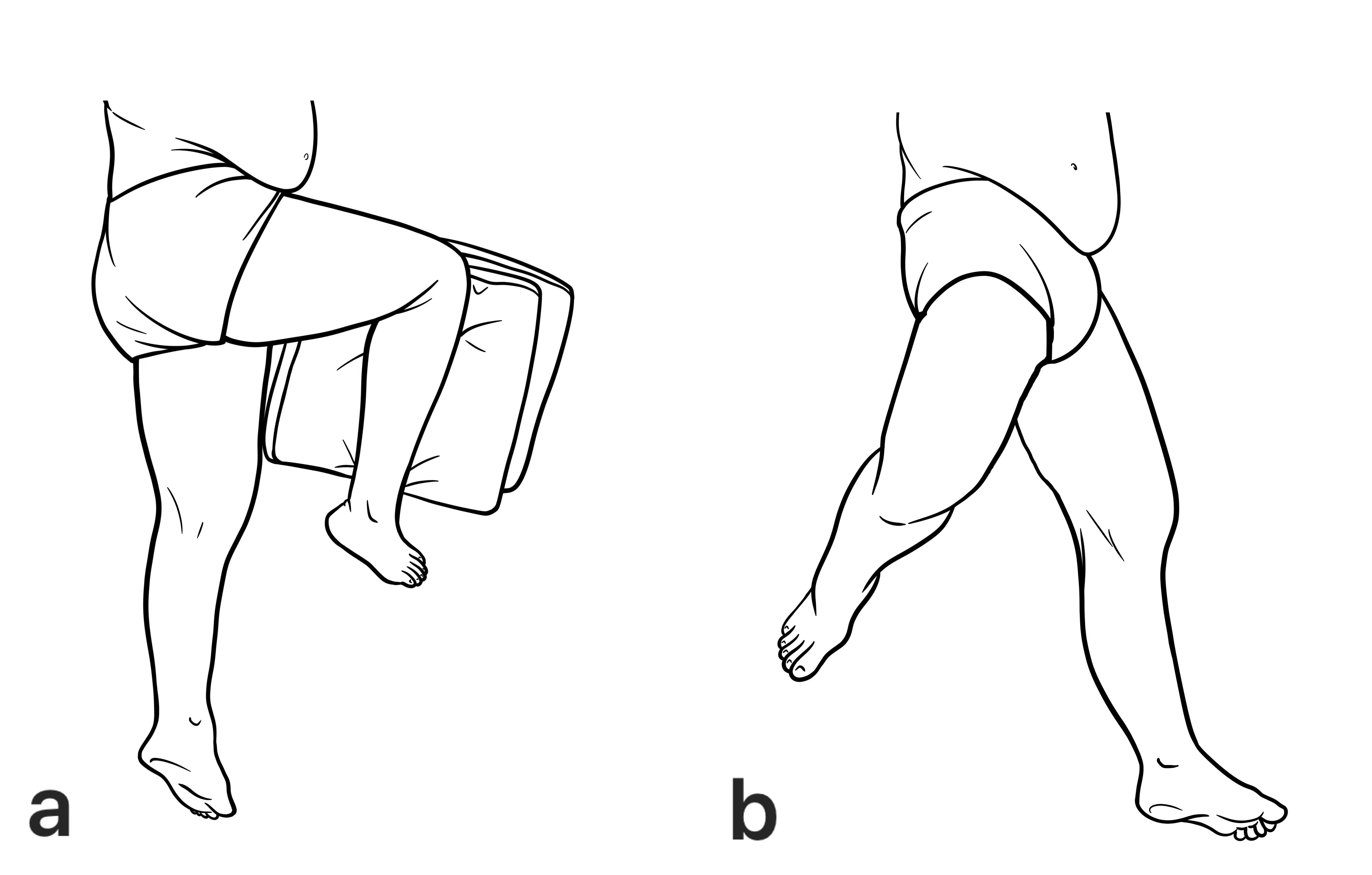


Fig. 2. categorization of lateral hip X-rays. a: Classic lateral view b: Modified lateral view c/d: In these figures, red and green lines represent unique skin patterns, while light red and light green areas indicate areas of overlap and increased density. It should be noted that comparing postoperative AP X-rays taken at the same time can also aid in making a judgment.


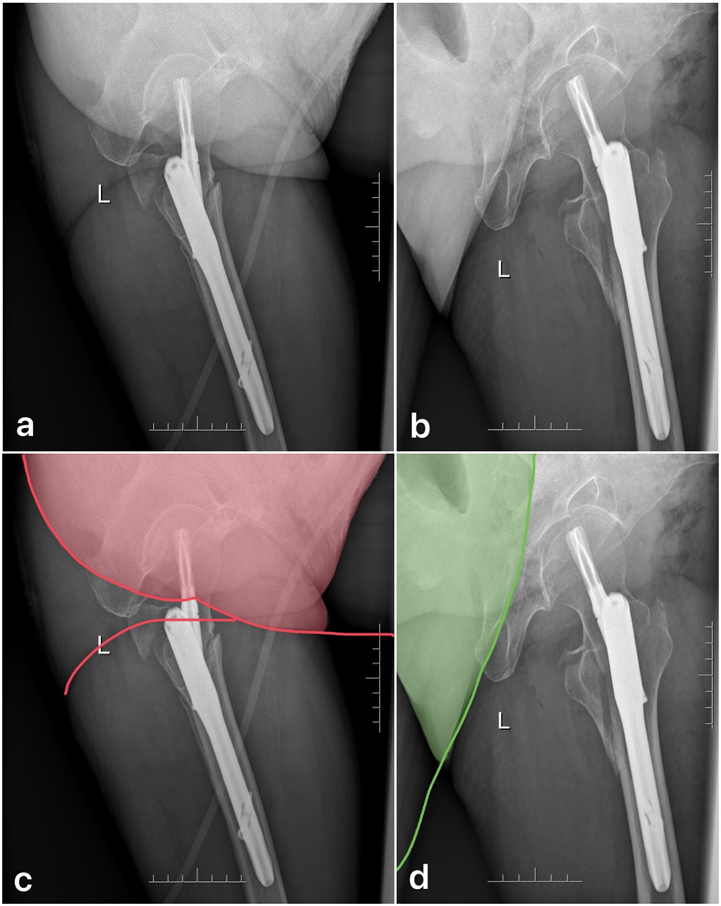


Fig. 3. a: CMNA = -12.9°. b:CMNA = +11.9°.c: "Ideal radiograph" showing CMNA = 1.4°. As the hip region is asymmetrical in the anteroposterior direction, a positive value was assigned when the helical blade was oriented in the same direction as the AP view, and a negative value was assigned when it was oriented in the opposite direction. d: The presence of the femoral bow and the obscuration of the femoral neck by the CMN make measurements such as NSA unreliable.

**
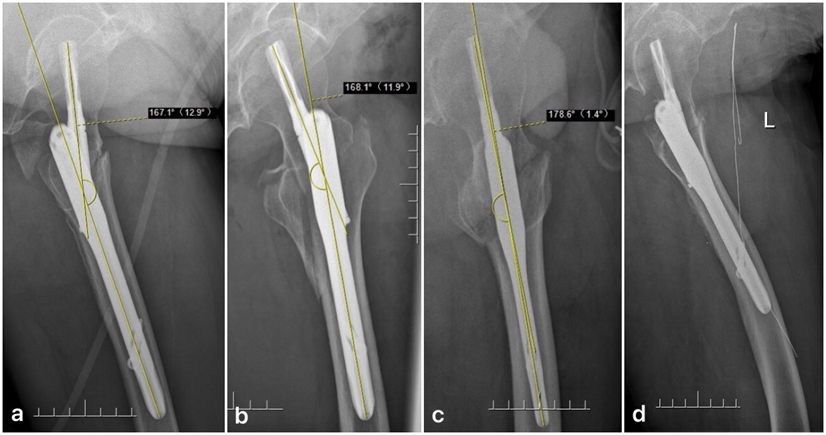
**

Fig.4 This image reveals the distribution of CMNA in two different shooting methods. It is noteworthy that the CMNA distribution in the classic lateral view was found to deviate from a normal distribution. Furthermore, the distribution of CMNA was observed to vary dramatically between the two X-ray imaging methods, which both differed significantly from the ideal lateral views of the femur head and neck (CMNA=0° and Normally distributed).

**
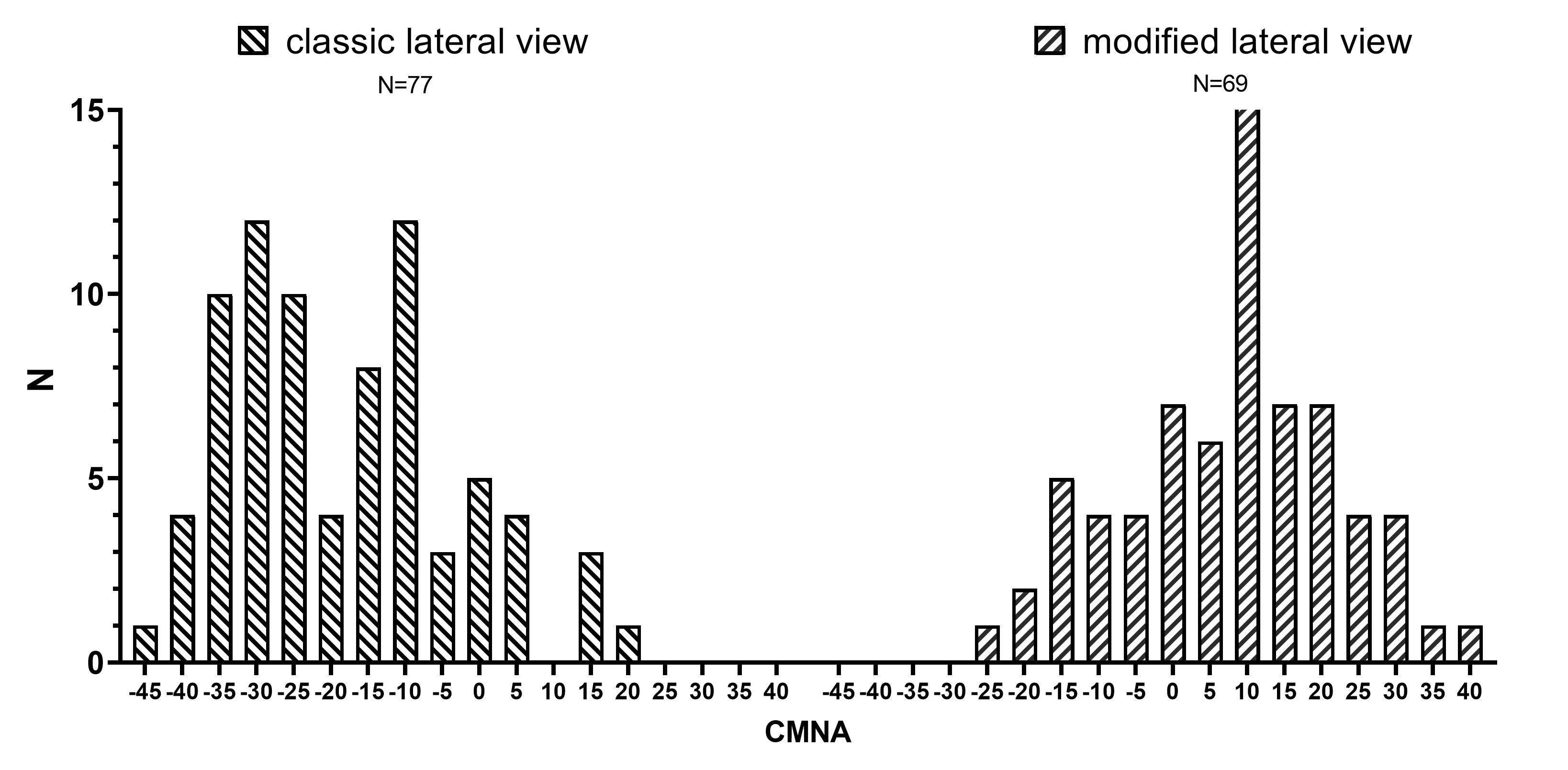
**

Fig. 5. a new head screw/blade position system. a: located at the posterior aspect. b: located at the middle aspect. c: located at the anterior aspect.

**
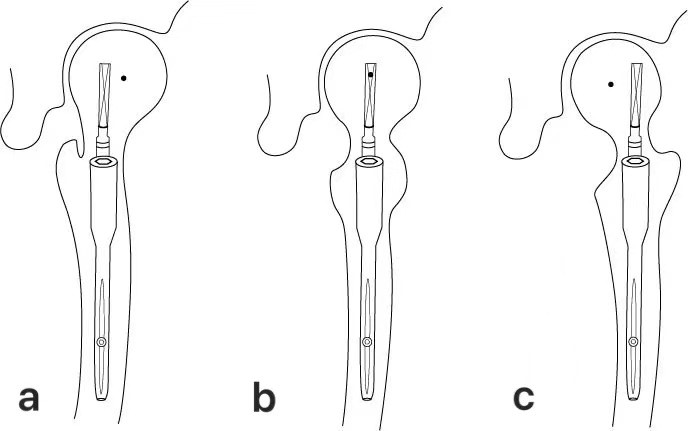
**

**Patient data involved in the study:**

**gender 1=Male 2=Female**

**side 1=Left 2=Right**

**type 1=classic lateral view 2= modified lateral view**

**AOclassification 1=A1 2=A2 3=A 3 4=Subtrochanteric**

**Naillength 1=Short Nail 2=Long Nail**

**All the raw hip image used and/or analysed during the current study are available from the corresponding author on reasonable request.**

| id | gender | age | side | type | AOclassification | daybeforesurgery | daysurgerytoXray | naillength | weight | height | BMI | CMNA1 | CMNA2 | CMNA3 | CMNA4 | CMNAmean |
| --- | --- | --- | --- | --- | --- | --- | --- | --- | --- | --- | --- | --- | --- | --- | --- | --- |
| 0001023417 | 2 | 80 | 1 | 1 | 2 | 4 | 4 | 1 | 45.00 | 150.00 | 20.00 | -6.6 | -5.9 | -5.9 | -5.6 | -6.0 |
| 0001029434 | 2 | 77 | 1 | 1 | 2 | 2 | 2 | 1 | 55.00 | 155.00 | 22.89 | 7.2 | 5.4 | 5.3 | 4.8 | 5.7 |
| 0001032940 | 2 | 63 | 2 | 1 | 4 | 1 | 1 | 2 | 56.00 | 154.00 | 23.61 | 7.2 | 7.4 | 7.4 | 7.5 | 7.4 |
| 0001033695 | 2 | 68 | 1 | 1 | 2 | 1 | 3 | 1 | 60.00 | 158.00 | 24.03 | -3.0 | -3.7 | -3.4 | -4.2 | -3.6 |
| 0001033753 | 2 | 83 | 1 | 2 | 2 | 1 | 2 | 1 | 50.00 | 155.00 | 20.81 | 36.8 | 36.3 | 37.4 | 37.2 | 36.9 |
| 0001035221 | 2 | 92 | 2 | 1 | 2 | 1 | 1 | 1 | 60.00 | 154.00 | 25.30 | -32.2 | -32.6 | -31.9 | -32.1 | -32.2 |
| 0001039027 | 1 | 84 | 1 | 1 | 3 | 16 | 4 | 2 | 50.00 | 163.00 | 18.82 | -28.3 | -28.4 | -28.3 | -28.3 | -28.3 |
| 0000830095 | 2 | 81 | 1 | 1 | 3 | 9 | 2 | 1 | 55.00 | 145.00 | 26.16 | -42.3 | -42.5 | -42.6 | -42.5 | -42.5 |
| 0001060317 | 2 | 75 | 2 | 1 | 2 | 1 | 2 | 1 | 41.00 | 150.00 | 18.22 | -32.8 | -32.7 | -33.1 | -34.1 | -33.2 |
| 0001069235 | 1 | 68 | 1 | 1 | 2 | 16 | 2 | 1 | 60.00 | 174.00 | 19.82 | -36.9 | -37.8 | -36.8 | -36.7 | -37.1 |
| 0001104848 | 1 | 75 | 1 | 1 | 2 | 3 | 2 | 1 | 60.00 | 165.00 | 22.04 | -8.4 | -7.9 | -7.6 | -8.1 | -8.0 |
| 0001108266 | 2 | 79 | 2 | 2 | 2 | 6 | 1 | 1 | 50.00 | 150.00 | 22.22 | 27.8 | 28.0 | 28.1 | 28.1 | 28.0 |
| 0001125527 | 1 | 58 | 2 | 2 | 3 | 3 | 2 | 2 |  |  |  | 10.3 | 9.8 | 9.8 | 9.7 | 9.9 |
| 0001144448 | 1 | 86 | 1 | 2 | 2 | 2 | 2 | 1 | 58.00 | 168.00 | 20.55 | -3.3 | -3.6 | -2.1 | -3.5 | -3.1 |
| 0001146310 | 2 | 82 | 1 | 1 | 2 | 2 | 2 | 1 | 55.00 | 156.00 | 22.60 | -17.4 | -17.5 | -17.8 | -17.2 | -17.5 |
| 0001124718 | 2 | 69 | 2 | 1 | 2 | 2 | 4 | 1 | 82.00 | 165.00 | 30.12 | -29.7 | -29.1 | -29.9 | -29.9 | -29.7 |
| 0001170406 | 2 | 85 | 1 | 1 | 2 | 6 | 1 | 1 |  |  |  | -38.3 | -37.9 | -38.0 | -38.3 | -38.1 |
| 0001184924 | 1 | 82 | 1 | 1 | 2 | 2 | 0 | 1 | 80.00 | 178.00 | 25.25 | 17.2 | 16.4 | 16.9 | 16.9 | 16.9 |
| 0001175234 | 2 | 77 | 2 | 2 | 2 | 27 | 2 | 1 |  |  |  | -18.9 | -18.2 | -18.3 | -18.3 | -18.4 |
| 0001194585 | 1 | 89 | 1 | 2 | 2 | 2 | 3 | 1 | 50.00 | 165.00 | 18.37 | 11.5 | 10.4 | 11.4 | 10.9 | 11.1 |
| 0000305945 | 1 | 72 | 1 | 1 | 2 | 0 | 2 | 1 | 67.50 | 170.00 | 23.36 | 6.7 | 6.3 | 6.4 | 6.2 | 6.4 |
| 0001208153 | 1 | 86 | 2 | 2 | 2 | 2 | 5 | 1 | 44.00 | 167.00 | 15.78 | -16.7 | -16.7 | -16.5 | -16.1 | -16.5 |
| 0001215073 | 1 | 81 | 2 | 2 | 2 | 2 | 3 | 1 | 65.00 | 165.00 | 23.88 | 13.5 | 13.1 | 13.5 | 13.6 | 13.4 |
| 0001216846 | 2 | 86 | 2 | 1 | 2 | 3 | 1 | 1 | 50.00 | 155.00 | 20.81 | -10.3 | -10.6 | -10.9 | -11.5 | -10.8 |
| 0001222757 | 2 | 67 | 1 | 1 | 2 | 2 | 2 | 1 |  |  |  | -27.2 | -27.2 | -27.4 | -27.9 | -27.4 |
| 0000908709 | 2 | 68 | 2 | 2 | 2 | 4 | 1 | 1 | 46.00 | 155.00 | 29.68 | 31.8 | 31.9 | 31.9 | 31.2 | 31.7 |
| 0001233858 | 2 | 82 | 1 | 2 | 1 | 13 | 1 | 1 | 60.00 | 155.00 | 24.97 | -10.2 | -10.5 | -9.7 | -9.8 | -10.1 |
| 0001239045 | 2 | 83 | 2 | 2 | 2 | 1 | 3 | 1 | 65.00 | 160.00 | 25.39 | 29.9 | 30.4 | 30.2 | 29.9 | 30.1 |
| 0001262369 | 2 | 88 | 1 | 1 | 3 | 2 | 1 | 2 |  |  |  | -30.4 | -30.2 | -29.3 | -30.4 | -30.1 |
| 0001270010 | 2 | 65 | 2 | 1 | 2 | 4 | 3 | 1 | 70.00 | 158.00 | 20.04 | -22.0 | -22.1 | -21.5 | -21.6 | -21.8 |
| 0001285862 | 1 | 72 | 2 | 1 | 2 | 0 | 5 | 1 | 60.00 | 170.00 | 20.76 | .0 | .0 | .0 | .0 | .0 |
| 0001295163 | 2 | 82 | 1 | 1 | 2 | 2 | 5 | 1 |  |  |  | -24.3 | -23.9 | -23.8 | -23.9 | -24.0 |
| 0001296561 | 1 | 65 | 1 | 1 | 3 | 2 | 2 | 2 | 60.00 | 172.00 | 20.28 | -9.9 | -8.1 | -7.9 | -10.3 | -9.1 |
| 0001311463 | 1 | 61 | 1 | 1 | 3 | 11 | 2 | 1 | 55.00 | 170.00 | 19.03 | -27.4 | -26.8 | -26.5 | -27.2 | -27.0 |
| 0001315562 | 1 | 73 | 2 | 1 | 2 | 1 | 2 | 1 | 46.00 | 170.00 | 15.92 | -26.8 | -26.6 | -27.5 | -27.3 | -27.1 |
| 0000236962 | 2 | 82 | 2 | 2 | 3 | 2 | 2 | 2 | 58.00 | 155.00 | 24.14 | 7.5 | 6.4 | 6.7 | 6.6 | 6.8 |
| 0001318838 | 1 | 71 | 1 | 2 | 2 | 1 | 2 | 1 | 80.00 | 180.00 | 24.69 | 7.0 | 7.2 | 6.9 | 6.9 | 7.0 |
| 0001321832 | 2 | 74 | 1 | 1 | 2 | 3 | 3 | 1 | 55.00 | 160.00 | 21.48 | -16.4 | -15.7 | -15.3 | -15.9 | -15.8 |
| 0001323634 | 2 | 73 | 1 | 1 | 2 | 5 | 0 | 1 | 49.00 | 150.00 | 21.78 | -12.9 | -11.6 | -12.5 | -12.2 | -12.3 |
| 0001325836 | 2 | 75 | 2 | 2 | 2 | 11 | 4 | 2 | 58.00 | 155.00 | 24.14 | 11.4 | 11.8 | 10.9 | 11.3 | 11.4 |
| 0001330936 | 2 | 74 | 1 | 2 | 2 | 21 | 1 | 1 | 48.00 | 155.00 | 19.98 | 17.9 | 17.5 | 17.9 | 18.1 | 17.9 |
| 0001334576 | 2 | 76 | 2 | 1 | 2 | 1 | 2 | 1 | 60.00 | 160.00 | 23.44 | -8.2 | -6.9 | -7.5 | -7.9 | -7.6 |
| 0001339958 | 2 | 84 | 2 | 1 | 3 | 2 | 3 | 2 | 53.00 | 155.00 | 22.06 | -9.4 | -9.5 | -9.6 | -9.5 | -9.5 |
| 0001340633 | 2 | 76 | 1 | 1 | 2 | 18 | 3 | 1 | 60.00 | 160.00 | 23.44 | -39.6 | -39.9 | -39.7 | -40.0 | -39.8 |
| 0001345908 | 2 | 77 | 1 | 2 | 2 | 5 | 2 | 1 | 60.00 | 148.00 | 27.39 | 41.1 | 41.2 | 40.6 | 40.8 | 40.9 |
| 0001348385 | 2 | 61 | 1 | 1 | 2 | 3 | 3 | 1 | 53.00 | 150.00 | 23.56 | -25.9 | -25.6 | -25.8 | -26.0 | -25.8 |
| 0001340200 | 1 | 69 | 2 | 2 | 2 | 20 | 5 | 1 | 75.00 | 175.00 | 24.49 | -23.5 | -22.0 | -22.2 | -21.7 | -22.4 |
| 0001315231 | 2 | 70 | 2 | 1 | 2 | 35 | 2 | 1 | 52.00 | 160.00 | 20.31 | -11.0 | -10.7 | -10.2 | -10.6 | -10.6 |
| 0000996507 | 2 | 71 | 1 | 2 | 1 | 1 | 2 | 1 | 53.00 | 165.00 | 19.47 | -7.1 | -7.2 | -7.1 | -8.1 | -7.4 |
| 0001382193 | 2 | 83 | 1 | 1 | 2 | 2 | 3 | 1 | 66.00 | 160.00 | 25.78 | -10.6 | -11.1 | -11.0 | -11.2 | -11.0 |
| 0001388313 | 1 | 75 | 2 | 2 | 2 | 2 | 2 | 1 | 60.00 | 170.00 | 20.76 | 12.9 | 12.1 | 13.2 | 13.1 | 12.8 |
| 0001388924 | 2 | 68 | 2 | 2 | 2 | 2 | 2 | 1 | 66.00 | 161.00 | 25.46 | -4.6 | -3.4 | -3.8 | -2.8 | -3.7 |
| 0001390304 | 2 | 69 | 2 | 1 | 2 | 3 | 3 | 1 | 37.50 | 155.00 | 15.61 | -15.3 | -15.2 | -15.5 | -15.1 | -15.3 |
| 0000044997 | 1 | 89 | 1 | 1 | 2 | 8 | 3 | 1 |  |  |  | 16.6 | 16.3 | 16.3 | 16.1 | 16.3 |
| 0001416170 | 2 | 86 | 2 | 1 | 2 | 1 | 2 | 1 |  |  |  | -21.6 | -21.0 | -22.6 | -21.1 | -21.6 |
| 0001417699 | 1 | 62 | 1 | 1 | 3 | 9 | 10 | 1 |  |  |  | -28.0 | -28.2 | -27.8 | -28.0 | -28.0 |
| 0001422309 | 2 | 84 | 1 | 1 | 2 | 6 | 2 | 1 | 55.00 | 155.00 | 22.89 | -30.9 | -30.7 | -30.1 | -30.7 | -30.6 |
| 0001433303 | 2 | 78 | 1 | 2 | 4 | 2 | 1 | 2 | 57.00 | 170.00 | 19.72 | 13.3 | 13.1 | 13.3 | 12.3 | 13.0 |
| 0001454701 | 2 | 69 | 1 | 1 | 2 | 4 | 3 | 1 | 55.00 | 163.00 | 20.70 | -32.3 | -32.5 | -31.9 | -32.6 | -32.3 |
| 0001528179 | 1 | 80 | 1 | 1 | 2 | 6 | 4 | 1 | 50.00 | 162.00 | 19.05 | 17.3 | 16.9 | 17.7 | 17.3 | 17.3 |
| 0001532027 | 2 | 72 | 2 | 2 | 2 | 9 | 2 | 1 | 50.00 | 155.00 | 20.81 | .0 | .0 | .0 | .0 | .0 |
| 0001555982 | 2 | 73 | 2 | 2 | 3 | 3 | 3 | 2 | 70.00 | 162.00 | 26.67 | 16.9 | 16.5 | 16.5 | 16.8 | 16.7 |
| 0001576593 | 2 | 82 | 1 | 1 | 2 | 6 | 2 | 1 | 60.00 | 160.00 | 23.44 | -19.2 | -18.8 | -19.1 | -19.5 | -19.2 |
| 0001571233 | 1 | 73 | 2 | 2 | 2 | 11 | 1 | 1 | 82.00 | 171.00 | 28.04 | -2.5 | -2.3 | -1.3 | -2.7 | -2.2 |
| 0001583260 | 1 | 83 | 1 | 2 | 2 | 5 | 2 | 1 | 66.00 | 169.00 | 23.11 | 14.0 | 14.6 | 14.0 | 14.6 | 14.3 |
| 0001584957 | 1 | 77 | 2 | 1 | 2 | 4 | 3 | 1 | 65.00 | 170.00 | 22.49 | .0 | .0 | .0 | .0 | .0 |
| 0000401277 | 1 | 73 | 2 | 1 | 2 | 8 | 2 | 2 |  |  |  | -16.5 | -16.2 | -16.3 | -16.7 | -16.4 |
| 0001591413 | 1 | 73 | 1 | 1 | 2 | 2 | 2 | 2 | 60.00 | 175.00 | 19.59 | -36.3 | -36.1 | -36.0 | -36.6 | -36.3 |
| 0001595850 | 1 | 67 | 1 | 1 | 1 | 4 | 2 | 1 | 55.00 | 157.00 | 22.31 | -16.7 | -16.3 | -16.2 | -16.0 | -16.3 |
| 0001601443 | 1 | 76 | 2 | 2 | 2 | 4 | 2 | 1 | 50.00 | 172.00 | 16.90 | -5.5 | -4.3 | -4.5 | -4.8 | -4.8 |
| 0001614446 | 2 | 78 | 1 | 1 | 2 | 0 | 2 | 1 | 75.00 | 165.00 | 27.55 | -12.1 | -12.3 | -12.2 | -11.8 | -12.1 |
| 0001623800 | 2 | 89 | 2 | 1 | 2 | 3 | 2 | 1 | 65.00 | 160.00 | 25.39 | -25.9 | -25.9 | -25.9 | -26.3 | -26.0 |
| 0001630310 | 2 | 90 | 1 | 2 | 2 | 2 | 2 | 1 | 50.00 | 150.00 | 22.22 | 20.9 | 19.9 | 20.4 | 20.4 | 20.4 |
| 0001666542 | 2 | 76 | 1 | 2 | 2 | 5 | 5 | 1 | 47.00 | 150.00 | 20.89 | 9.7 | 8.8 | 9.9 | 9.1 | 9.4 |
| 0001189279 | 2 | 73 | 2 | 2 | 2 | 2 | 1 | 1 |  |  |  | 22.8 | 22.7 | 22.2 | 22.4 | 22.5 |
| 0001706622 | 1 | 90 | 2 | 2 | 2 | 4 | 2 | 1 | 55.00 | 165.00 | 20.20 | 11.7 | 12.1 | 12.4 | 11.7 | 12.0 |
| 0001719897 | 2 | 71 | 1 | 2 | 2 | 1 | 1 | 1 | 70.00 | 165.00 | 25.71 | .0 | .0 | .0 | .0 | .0 |
| 0001721699 | 1 | 86 | 1 | 2 | 2 | 1 | 2 | 1 | 50.00 | 176.00 | 16.14 | 17.7 | 18.1 | 17.9 | 18.4 | 18.0 |
| 0001744727 | 2 | 64 | 1 | 2 | 2 | 4 | 1 | 1 | 50.00 | 164.00 | 18.59 | 11.9 | 10.6 | 11.3 | 10.9 | 11.2 |
| 0001654418 | 2 | 67 | 1 | 2 | 1 | 0 | 2 | 1 | 70.00 | 165.00 | 25.71 | 24.0 | 25.0 | 25.6 | 25.2 | 25.0 |
| 0001757740 | 2 | 82 | 1 | 1 | 2 | 4 | 3 | 1 | 65.00 | 156.00 | 26.71 | -31.7 | -30.8 | -31.0 | -30.4 | -31.0 |
| 0001758813 | 2 | 92 | 1 | 1 | 2 | 4 | 2 | 1 | 37.50 | 160.00 | 14.65 | -6.9 | -7.3 | -6.7 | -6.5 | -6.9 |
| 0001206651 | 2 | 72 | 2 | 2 | 2 | 4 | 3 | 1 | 60.00 | 160.00 | 23.44 | .0 | .0 | .0 | .0 | .0 |
| 0001766753 | 2 | 71 | 1 | 2 | 2 | 9 | 2 | 1 | 60.00 | 160.00 | 23.44 | 9.2 | 8.6 | 9.1 | 9.5 | 9.1 |
| 0002005425 | 2 | 67 | 1 | 2 | 3 | 8 | 2 | 2 | 50.00 | 150.00 | 22.22 | 29.7 | 29.4 | 29.6 | 29.4 | 29.5 |
| 0002020474 | 2 | 83 | 2 | 1 | 2 | 9 | 4 | 1 | 42.00 | 145.00 | 19.98 | -43.6 | -42.2 | -42.6 | -42.9 | -42.8 |
| 0002023126 | 1 | 58 | 1 | 2 | 2 | 0 | 2 | 1 | 80.00 | 173.00 | 26.73 | -8.2 | -7.5 | -7.5 | -7.5 | -7.7 |
| 0002022386 | 2 | 71 | 1 | 1 | 2 | 2 | 2 | 1 | 46.00 | 155.00 | 19.15 | -41.4 | -38.6 | -41.0 | -40.7 | -40.4 |
| 0002038981 | 2 | 81 | 1 | 2 | 2 | 3 | 2 | 1 | 73.00 | 160.00 | 28.52 | 7.2 | 7.4 | 8.1 | 8.0 | 7.7 |
| 0002007742 | 2 | 58 | 2 | 1 | 1 | 4 | 2 | 1 | 43.00 | 160.00 | 16.80 | 5.2 | 6.2 | 5.9 | 5.0 | 5.6 |
| 0001401811 | 2 | 83 | 2 | 2 | 2 | 3 | 2 | 1 | 35.00 | 150.00 | 15.56 | 19.5 | 20.0 | 19.8 | 19.6 | 19.7 |
| 0002075779 | 1 | 60 | 2 | 2 | 2 | 4 | 2 | 1 |  |  |  | -17.7 | -17.0 | -17.2 | -17.4 | -17.3 |
| 0002077084 | 2 | 72 | 2 | 2 | 2 | 4 | 2 | 1 | 70.00 | 165.00 | 25.71 | -12.5 | -12.8 | -13.1 | -13.2 | -12.9 |
| 0002070871 | 2 | 93 | 1 | 2 | 3 | 15 | 2 | 1 | 55.00 | 153.00 | 23.50 | 25.9 | 25.7 | 25.7 | 26.1 | 25.9 |
| 0002084788 | 2 | 65 | 2 | 2 | 2 | 1 | 2 | 1 | 67.50 | 155.00 | 28.10 | -22.9 | -22.8 | -23.6 | -22.6 | -23.0 |
| 0002074500 | 2 | 81 | 1 | 2 | 2 | 5 | 2 | 1 | 70.00 | 165.00 | 25.71 | 22.3 | 22.2 | 22.5 | 22.5 | 22.4 |
| 0002082539 | 1 | 69 | 1 | 1 | 2 | 20 | 3 | 1 | 60.00 | 176.00 | 19.37 | -7.8 | -7.9 | -7.7 | -7.6 | -7.8 |
| 0002096321 | 2 | 74 | 2 | 2 | 4 | 14 | 5 | 2 | 75.00 | 167.00 | 26.89 | 11.5 | 11.9 | 11.5 | 11.4 | 11.6 |
| 0002102648 | 1 | 63 | 1 | 1 | 2 | 4 | 1 | 1 | 55.00 | 164.00 | 20.45 | .0 | .0 | 2.7 | 2.6 | 1.3 |
| 0000145821 | 2 | 72 | 2 | 1 | 4 | 2 | 3 | 2 | 52.50 | 162.00 | 20.00 | -32.6 | -33.4 | -32.7 | -32.8 | -32.9 |
| 0002128436 | 1 | 82 | 2 | 2 | 2 | 3 | 3 | 2 | 55.00 | 162.00 | 20.96 | 8.2 | 8.0 | 7.9 | 7.4 | 7.9 |
| 0002133777 | 2 | 79 | 1 | 1 | 2 | 2 | 1 | 1 | 50.00 | 150.00 | 22.22 | -30.4 | -27.2 | -30.0 | -30.1 | -29.4 |
| 0002139944 | 1 | 59 | 1 | 1 | 2 | 3 | 2 | 1 | 50.00 | 164.00 | 18.59 | -24.8 | -24.3 | -24.4 | -24.7 | -24.6 |
| 0000648042 | 1 | 83 | 1 | 1 | 2 | 19 | 3 | 1 | 62.00 | 172.00 | 20.96 | .0 | .0 | -1.4 | -1.8 | -.8 |
| 0002144565 | 2 | 85 | 2 | 1 | 2 | 2 | 2 | 1 | 50.00 | 160.00 | 19.53 | -31.6 | -31.4 | -31.7 | -31.4 | -31.5 |
| 0002157239 | 1 | 78 | 2 | 2 | 2 | 14 | 0 | 1 |  |  |  | 23.0 | 23.2 | 23.0 | 23.1 | 23.1 |
| 0002180453 | 2 | 88 | 2 | 1 | 3 | 4 | 2 | 1 | 80.00 | 150.00 | 35.56 | -31.3 | -31.1 | -31.7 | -31.0 | -31.3 |
| 0002184239 | 2 | 86 | 1 | 1 | 2 | 4 | 1 | 1 | 40.00 | 155.00 | 16.65 | -37.4 | -36.6 | -36.7 | -37.3 | -37.0 |
| 0002197176 | 1 | 63 | 1 | 2 | 2 | 2 | 2 | 1 | 82.00 | 175.00 | 26.78 | 10.0 | 8.7 | 9.0 | 8.5 | 9.1 |
| 0002223143 | 2 | 75 | 1 | 1 | 2 | 1 | 1 | 1 |  |  |  | -36.3 | -36.4 | -36.2 | -36.3 | -36.3 |
| 0002223566 | 1 | 82 | 2 | 2 | 2 | 3 | 2 | 1 | 60.00 | 165.00 | 22.04 | 15.5 | 15.3 | 14.7 | 14.9 | 15.1 |
| 0002286678 | 2 | 74 | 1 | 2 | 3 | 2 | 1 | 1 | 62.50 | 152.00 | 27.05 | .0 | .0 | 1.8 | .3 | .5 |
| 0002305721 | 1 | 66 | 1 | 2 | 2 | 2 | 2 | 1 | 75.00 | 180.00 | 23.15 | 7.2 | 3.3 | 3.7 | 4.9 | 4.8 |
| 0002309288 | 2 | 66 | 2 | 1 | 3 | 9 | 2 | 1 | 42.50 | 158.00 | 17.02 | 17.7 | 17.6 | 17.5 | 17.3 | 17.5 |
| 0002318690 | 1 | 70 | 2 | 2 | 2 | 2 | 1 | 1 | 84.00 | 170.00 | 29.07 | 8.4 | 8.2 | 8.5 | 8.3 | 8.4 |
| 0002356060 | 2 | 61 | 1 | 2 | 3 | 8 | 2 | 1 | 40.00 | 160.00 | 15.62 | 19.6 | 19.5 | 18.8 | 19.7 | 19.4 |
| 0000994243 | 2 | 72 | 2 | 1 | 2 | 3 | 1 | 1 | 45.00 | 155.00 | 18.73 | -8.7 | -9.0 | -9.4 | -9.2 | -9.1 |
| 0002423087 | 1 | 75 | 2 | 1 | 3 | 2 | 2 | 2 | 63.00 | 175.00 | 20.57 | -32.2 | -33.1 | -32.5 | -32.7 | -32.6 |
| 0000038449 | 1 | 93 | 1 | 2 | 2 | 3 | 1 | 1 | 60.00 | 169.00 | 21.00 | -17.2 | -16.5 | -16.6 | -16.6 | -16.7 |
| 0002460616 | 2 | 71 | 2 | 2 | 2 | 4 | 2 | 1 | 55.00 | 150.00 | 24.44 | -12.0 | -11.8 | -11.5 | -11.3 | -11.7 |
| 0000090363 | 2 | 72 | 1 | 2 | 2 | 3 | 2 | 1 | 75.00 | 159.00 | 29.67 | 12.3 | 10.5 | 10.2 | 10.0 | 10.8 |
| 0002538057 | 2 | 74 | 1 | 1 | 2 | 6 | 2 | 1 | 60.00 | 158.00 | 24.03 | -33.9 | -32.9 | -32.9 | -33.8 | -33.4 |
| 0002553014 | 2 | 69 | 2 | 2 | 2 | 2 | 1 | 1 | 55.00 | 158.00 | 22.03 | 4.4 | 4.5 | 3.0 | 3.1 | 3.8 |
| 0002558558 | 2 | 88 | 2 | 2 | 3 | 5 | 2 | 1 | 70.00 | 158.00 | 28.04 | 10.6 | 10.6 | 9.9 | 10.1 | 10.3 |
| 0002571249 | 2 | 76 | 1 | 1 | 2 | 2 | 2 | 1 | 77.00 | 167.00 | 27.61 | -33.1 | -32.4 | -32.5 | -33.0 | -32.8 |
| 0001397682 | 2 | 78 | 1 | 1 | 3 | 4 | 1 | 1 | 40.00 | 126.00 | 25.20 | -15.6 | -15.1 | -15.1 | -15.0 | -15.2 |
| 0000851133 | 2 | 81 | 2 | 1 | 2 | 8 | 1 | 1 | 52.00 | 158.00 | 20.83 | -29.5 | -29.3 | -29.7 | -29.4 | -29.5 |
| 0002588773 | 1 | 76 | 1 | 1 | 2 | 2 | 2 | 1 | 72.00 | 175.00 | 23.51 | -17.3 | -16.8 | -16.7 | -17.0 | -17.0 |
| 0002591657 | 1 | 86 | 1 | 2 | 2 | 2 | 2 | 1 | 60.00 | 168.00 | 21.26 | 3.2 | 3.3 | 2.7 | 3.0 | 3.1 |
| 0002604613 | 1 | 72 | 2 | 1 | 4 | 3 | 2 | 2 | 60.00 | 173.00 | 20.05 | -17.0 | -17.2 | -17.4 | -17.3 | -17.2 |
| 0002609510 | 2 | 87 | 2 | 1 | 3 | 3 | 1 | 1 | 45.00 | 150.00 | 20.00 | -25.5 | -25.3 | -26.2 | -25.7 | -25.7 |
| 0002616714 | 1 | 61 | 1 | 1 | 2 | 4 | 1 | 1 |  |  |  | -27.5 | -27.5 | -27.2 | -27.1 | -27.3 |
| 0002622010 | 2 | 72 | 2 | 2 | 1 | 3 | 1 | 1 | 50.00 | 158.00 | 20.03 | -7.9 | -8.2 | -7.8 | -7.6 | -7.9 |
| 0002628246 | 2 | 79 | 1 | 1 | 3 | 5 | 1 | 1 |  |  |  | -25.3 | -24.8 | -24.7 | -24.7 | -24.9 |
| 0002620237 | 2 | 63 | 2 | 2 | 2 | 18 | 2 | 1 | 50.00 | 160.00 | 19.53 | 7.7 | 8.7 | 8.7 | 8.6 | 8.4 |
| 0002632923 | 2 | 65 | 2 | 2 | 1 | 2 | 1 | 1 | 59.00 | 160.00 | 23.05 | 14.3 | 14.3 | 13.8 | 13.7 | 14.0 |
| 0000867090 | 2 | 81 | 2 | 1 | 2 | 9 | 3 | 1 | 80.00 | 158.00 | 32.05 | -35.6 | -35.7 | -34.8 | -34.2 | -35.1 |
| 0002696007 | 2 | 80 | 1 | 2 | 2 | 4 | 1 | 1 | 60.00 | 165.00 | 22.04 | -14.0 | -14.2 | -13.9 | -14.2 | -14.1 |
| 0002697513 | 1 | 83 | 1 | 1 | 2 | 3 | 2 | 1 | 60.00 | 170.00 | 20.76 | -8.5 | -8.2 | -7.7 | -7.5 | -8.0 |
| 0001527700 | 1 | 86 | 1 | 1 | 2 | 1 | 21 | 1 |  |  |  | -20.8 | -20.8 | -19.9 | -20.1 | -20.4 |
| 0002728492 | 1 | 83 | 1 | 2 | 2 | 2 | 1 | 1 | 64.00 | 170.00 | 22.15 | 1.4 | 2.1 | 2.3 | 2.7 | 2.1 |
| 0002729860 | 2 | 81 | 2 | 2 | 3 | 2 | 3 | 1 | 65.00 | 160.00 | 25.39 | 2.4 | 3.4 | 3.5 | 3.8 | 3.3 |
| 0001745827 | 1 | 78 | 1 | 2 | 2 | 2 | 1 | 1 | 42.50 | 165.00 | 15.61 | -1.3 | -2.0 | -2.3 | -2.4 | -2.0 |
| 0002759468 | 2 | 80 | 2 | 1 | 2 | 7 | 1 | 1 | 60.00 | 155.00 | 24.97 | .8 | .0 | 1.2 | 1.2 | .8 |
| 0002761294 | 2 | 75 | 2 | 2 | 2 | 5 | 1 | 1 | 55.00 | 150.00 | 24.44 | 13.5 | 12.1 | 12.0 | 11.7 | 12.3 |
| 0002789820 | 1 | 89 | 2 | 2 | 2 | 3 | 1 | 1 | 45.00 | 165.00 | 16.53 | 21.0 | 21.3 | 21.9 | 21.4 | 21.4 |
